# The epidemiology, clinical presentation and treatment outcomes in CNS actinomycosis: a systematic review of reported cases

**DOI:** 10.1186/s13023-023-02744-z

**Published:** 2023-06-02

**Authors:** Durga Shankar Meena, Deepak Kumar, Mukesh Sharma, Manika Malik, Akshatha Ravindra, N. Santhanam, Gopal Krishana Bohra, Mahendra Kumar Garg

**Affiliations:** 1 Department of Internal Medicine (Infectious Diseases), All India Institute of Medical Sciences, Jodhpur, 342005 India; 2 Department of Microbiology, Dr. S.N. Medical College, Jodhpur, India

**Keywords:** CNS infections, Actinomycosis, Brain abscess, Penicillin, Sulphur granules

## Abstract

**Background:**

CNS actinomycosis is a rare chronic suppurative infection with non-specific clinical features. Diagnosis is difficult due to its similarity to malignancy, nocardiosis and other granulomatous diseases. This systematic review aimed to evaluate the epidemiology, clinical characteristics, diagnostic modalities and treatment outcomes in CNS actinomycosis.

**Methods:**

The major electronic databases (PubMed, Google Scholar, and Scopus) were searched for the literature review by using distinct keywords: "CNS" or "intracranial" or "brain abscess" or "meningitis" OR "spinal" OR "epidural abscess" and "actinomycosis." All cases with CNS actinomycosis reported between January 1988 to March 2022 were included.

**Results:**

A total of 118 cases of CNS disease were included in the final analysis. The mean age of patients was 44 years, and a significant proportion was male (57%). *Actinomycosis israelii* was the most prevalent species (41.5%), followed by *Actinomyces meyeri* (22.6%). Disseminated disease was found in 19.5% of cases. Most commonly involved extra-CNS organs are lung (10.2%) and abdomen (5.1%). Brain abscess (55%) followed by leptomeningeal enhancement (22%) were the most common neuroimaging findings. Culture positivity was found in nearly half of the cases (53.4%). The overall case-fatality rate was 11%. Neurological sequelae were present in 22% of the patients. On multivariate analysis, patients who underwent surgery with antimicrobials had better survival (adjusted OR 0.14, 95% CI 0.04–0.28, *p *value 0.039) compared to those treated with antimicrobials alone.

**Conclusion:**

CNS actinomycosis carries significant morbidity and mortality despite its indolent nature. Early aggressive surgery, along with prolonged antimicrobial treatment is vital to improve outcomes.

**Supplementary Information:**

The online version contains supplementary material available at 10.1186/s13023-023-02744-z.

## Background

Actinomycosis is a rare, subacute to chronic granulomatous infection caused by gram-positive anaerobic Actinomyces species [1]. The Actinomyces genus of the family Actinomycetaceae currently comprises around 42 species [1–3]. Most infections are caused by *Actinomyces israelii* and *A. gerencseriae* [4]. Actinomyces are non-spore-forming, non-acid-fast, filamentous microorganisms which are commensals in the human oropharynx (found in gingival crevices, dental plaques, tonsillar crypts), gastrointestinal and genitourinary flora [5, 6]. These organisms usually manifest as a slowly progressive, mass-like lesion which ultimately forms draining sinus tracts [6]. Actinomycosis is a great masquerader that can mimic malignancy and other indolent infections like fungal, nocardiosis and mycobacterial diseases. Of note, later two genera belong to the same order of Actinomycetales [7]. The Cervicofacial disease is the most common presentation of actinomycosis (around 50% of the cases), followed by abdominal (20%) and thoracic disease (15–20%) [5, 8–10].

Of note, actinomycosis is usually described as an indolent infection with protean manifestations; however, CNS involvement is the most severe form of actinomycosis [11]. CNS actinomycosis is a rare disease with an exact incidence that is difficult to ascertain due to the rarity of the disease. The majority of data is available as anecdotal evidence in the form of case reports and expert opinions. Friedman et al. performed the first systematic review of cases with CNS actinomycosis in 1937 [12]. However, there were a lot of cases where no differentiation was made between nocardiosis and actinomycosis. In 1964, Bolton et al. reviewed 17 cases with CNS actinomycosis [13]. The first comprehensive systematic review was published by Smego et al. in 1987 [14], which included 70 cases of CNS actinomycosis. To better understand the clinical spectrum and various predictors of outcome, we performed an updated systematic review of CNS actinomycosis and analyzed the cases reported from 1988 to 2022.

## Methods

### Protocol and registration

This systematic review is performed in accordance with the Preferred Reporting Items for Systematic Reviews and Meta-Analyses (PRISMA) statement (Additional file 1: S1) [15] and is registered in the PROSPERO online database (PROSPERO Identifier: CRD42022320661).

### Search strategy and information sources

We performed systematic searches of the literature to identify all the reported cases with CNS manifestations due to actinomycosis. A literature search was performed by using different electronic databases of the English literature (PubMed/Medline, Google Scholar and Scopus). We identified the published data (case reports and case series) reported between January 1988 to May 2022. The search terms for our review were: "CNS" or "intracranial" or "brain abscess" or "meningitis" OR "spinal" OR "epidural abscess" and "actinomycosis" in different combinations (Additional file 1: s2).

### Study selection (case definition and inclusion criteria)

We included 118 cases of CNS actinomycosis in this systematic review. The cases meeting the following criteria were included in the final analysis: a). Cases with isolation of actinomycosis from culture of a sterile site (CSF, abscess, tissue sample), or b). Demonstration of the gram-positive, non-acid-fast, non-spore-forming filamentous organism with characteristics sulphur granules with the compatible clinical syndrome, or c). Identification of actinomycosis species by molecular methods (e.g., 16S rRNA sequencing) with a compatible Clinico-radiological syndrome.

We included all cases with detailed documentation of clinical presentation, diagnostic methods, treatment and outcomes. The antimicrobial treatment in actinomycosis is usually prolonged (3 to 12 months, depending on the organ involved); thus, only cases with a follow-up of at least three months after the commencement of antimicrobial therapy were included in the surviving group. [14, 16, 17]. Review articles, editorials, and conference papers/posters were excluded. Patients who received corticosteroids (≥ 10 mg/day for at least four weeks), solid organ/stem cell transplant recipients, and long-term immunosuppressants were considered immunocompromised. Cases with evidence of hematogenous spread (actinomycosis bacteremia) or the involvement of distant sites (non-contiguous sites) like pulmonary, abdomen or pelvic were defined as disseminated actinomycosis. Cervicofacial actinomycosis includes the involvement of upper or lower mandible (osteomyelitis), cheek, chin, and submaxillary ramus and angle. Duration of illness was defined by the time since the patient developed symptoms until admission or the first presentation in clinic.

### Data extraction and qualitative assessment

Two authors (DSM and MM) independently extracted the data from the selected literature. The online systematic review software (Covidence systematic review software, Veritas Health Innovation, Melbourne, Australia) was used for the extraction of data. The following data were extracted: clinical history, comorbidities/risk factors, laboratory diagnosis, treatment modalities and outcome with follow-up (including residual disease or relapse). The disagreement between the authors was reconciled by the discussion and consensus with the other reviewer authors (DK, MS, AR). To reduce the inherent bias associated with case reports, we used the standardized critical appraisal tool proposed by the Joanna Briggs Institute (JBI) [18, 19]. The JBI critical appraisal checklist is provided in the supplementary material (Additional file 1: s3).

### Statistical analysis

Data analysis was conducted by using SPSS software, version 20.0 (IBM Corp, Armonk, NY). All descriptive data were summarised and tabulated with continuous variables in the form of mean ± standard deviation, median (with interquartile range), and categorical variables in the form of a number (percentages). Categorical variables were analyzed using Pearson’s chi-square test, and continuous variables were analyzed by Student’s t-test. Univariate regression analysis was performed to determine the various clinical predictors of mortality (age, gender, clinical presentation, type of actinomycosis species, immunocompromised state, and treatment modalities). Variables on univariate analysis showing significant correlation (*p *value ≤ 0.1) were selected for multivariate regression analysis. A *p *value < 0.05 was considered to indicate statistical significance. The multivariate results were presented as odds ratio and their 95% confidence intervals (CIs).

## Results

**Fig. 1:**
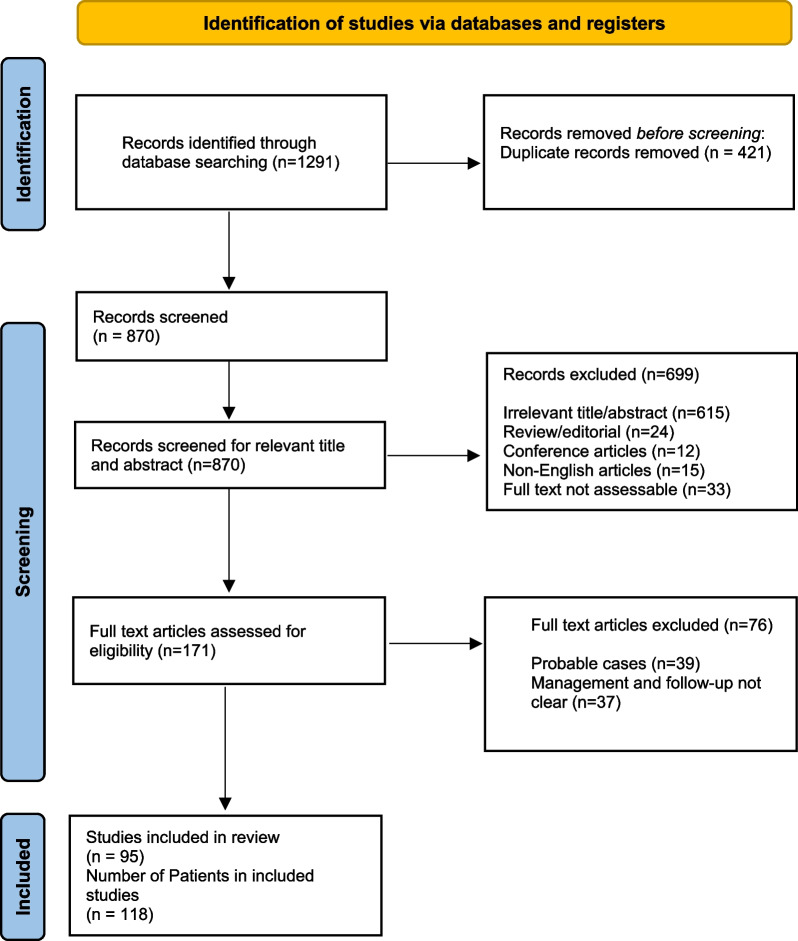
Flow chart of articles selection according to PRISMA guideline

The initial database search revealed a total of 1291 case records that were analyzed for the final inclusion. After removing duplicate records, 870 records were assessed for further inclusion. After the removal of ineligible articles, a total of 118 patients with CNS actinomycosis (95 articles) were analyzed in this systematic review (Fig. 1). Out of 118 patients, 29 were from a case series, and the remaining 89 were individual data. The reference list of all reported cases in this review is given in supplementary material (Additional file 1: s4).

### Patients’ characteristics

This systematic review identified a total of 118 cases of CNS actinomycosis. The median age of the patients was 45 years. Cases have been reported from 2 months to 90 years of age. The proportion of male patients was 57%. Most cases were reported from Asia (49%), followed by the USA and Europe (Table 1). We found only three cases reported from African countries.

**Table 1:** Clinical and Demographic characteristics of patients with CNS Actinomycosis

Characteristics	No. (%)
**Age (years)**	
Mean age ± SD	44 ± 20.6
Median age	45
Range	2–90
**Gender (N = 118)**	
Male	67 (56.8)
Female	51 (43.2)
**Geographical distribution (N = 118)**	
Asia	58 (49.2)
America	33 (28)
Europe	24 (20.3)
Africa	3 (2.5)
Case fatality	13/118 (11)
**Type of Actinomycosis species identified (N = 53)**	
*Actinomyces israelii*	22 (41.5)
*Actinomyces meyeri*	12 (22.6)
*Actinomyces viscosus*	6 (11.3)
*Actinomyces neuii*	4 (7.5)
*Actinomyces turicensis*	3 (5.7)
*Actinomyces odontolyticus*	3 (5.7)
*Actinomyces georgiae*	1 (1.9)
*Actinomyces naeslundii*	1 (1.9)
*Actinomyces oris*	1 (1.9)
Species not identified	65 (55)
Disseminated actinomycosis	23/118 (19.5)
Immunocompromised	15 (12.7)

### Clinical features, risk factors and comorbidities

Focal neurological deficits were the most common clinical presentation in CNS actinomycosis (65.3%). The other common presentations were headache, fever, and altered sensorium (Table 2). Meningeal signs were present in 21% of the patients. The mean duration of symptoms (time from the onset of disease to the first presentation in hospital) was 82 days in CNS disease, which indicates the indolent nature of the illness. The cervicofacial region was the most common other site involved in CNS actinomycosis due to contiguous spread of infection (Table 1). Of note, disseminated actinomycosis infection was reported in 19.5% (n = 23) of the cases (Table 1). In these disseminated cases, the other manifestations were pulmonary disease, abdominal actinomycosis and osteomyelitis (10.2%, 5.1% and 4.2%, respectively). Notwithstanding, actinomycosis bacteremia was found in 4 (3.4%) of the cases in this review [19–22]. One of these patients also had features of endocarditis as a clinical spectrum of disseminated actinomycosis [22]. All of these patients presented acutely (within 14 days of onset of symptoms). Isolated CNS disease was found in 80% (n = 95) of the patients.

**Table 2:** Clinical features in patients presented with CNS actinomycosis

Variables	N (%)
**Mean duration of symptoms**	82 days
Focal deficits^#^	77 (65.3)
Headache	60 (50.1)
Fever	51 (43.2)
LOC/altered sensorium	30 (25.4)
Meningeal signs	25 (21.2)
Seizure	19 (16.1)
Cranial nerve involvement	13 (11)
Other systematic features	19 (16.1)
Disseminated disease	23 (19.5)
**Other site involvement**	
Cervicofacial Actinomycosis	15 (12.7)
Pulmonary Actinomycosis	12 (10.2)
Abdominal Actinomycosis	6 (5.1)
Osteomyelitis	5 (4.2)
Actinomycosis bacteraemia	4 (3.4)
Skin	2 (1.7)

^#^focal deficits include limb weakness, speech abnormality, cerebellar sign, gait abnormality, visual disturbances

In this review, we also searched for various immunodeficient states, risk factors, and comorbidities associated with CNS actinomycosis. Only 12.7% of patients were immunocompromised (Table 1). Among predisposing factors, a history of dental procedures was the most frequent (in 15% of the patients, Table 3). Dental infections (active or in the recent past), head trauma/surgery, and ear infections were the other predisposing factors for CNS actinomycosis (Table 3). Of note, we could not find any risk factors or immunodeficiency state in 49 patients (41.5%). We also compared the clinical presentation of disseminated disease with isolated CNS actinomycosis. The mean age of disseminated cases was significantly increased compared to isolated CNS cases (57 vs 41 years, p =  < 0.003). Furthermore, the duration of illness prior to presentation was shorter in disseminated cases (44 vs 92 days). The proportion of immunodeficient patients was significantly higher in disseminated cases than in isolated CNS cases (23% vs 9.8%, p = 0.02).

**Table 3:** Various predisposing factors and comorbidities in patients with CNS Actinomycosis

Predisposing conditions	N (%)
Dental procedure/tooth extraction	18 (15.3)
Dental infections	17 (14.4)
Head trauma/past surgery	14 (11.9)
Otitis media/mastoiditis	13 (11)
Corticosteroids/other immunosuppressants	11 (9.3)
Chronic alcoholism	10 (8.5)
Diabetes mellitus	8 (6.8)
Malignancy	8 (6.8)
Ischemic heart disease	9 (7.6)
Chronic obstructive pulmonary disease	5 (4.2)
Autoimmune diseases	4 (3.4)
Chronic kidney disease	3 (2.5)
Chronic liver disease	2 (1.7)

### Methods of diagnosis (laboratory and radiological characteristics and species identification)

In most patients, the diagnosis of CNS disease was made by characteristic histopathological findings and culture. In 55% of patients, actinomycosis was identified in culture. The most common specimen for culture was pus aspirated from a brain abscess (Table 4). CSF (cerebrospinal fluid) culture positivity was found in nearly 17% of the patients. Actinomycosis was isolated from blood in four patients. CSF cytology report was available in only 19 patients in this review. More than two-thirds (68%) of patients showed a neutrophilic pleocytosis with a median protein of 112 mg/dl (Table 4). The molecular diagnosis was established in 12 cases by MALDI-TOF (Matrix-Assisted Laser Desorption/Ionization-Time of Flight), PCR and 16S RNA sequencing methods. The species identification was possible in only 53 cases (Table 1). *Actinomyces israelii* was the most common species causing CNS disease, followed by *A. meyeri* and *A. viscosus* (41.5%, 22.6% and 11.3%, respectively). *A. neuii, A. turicensis*, *A. odontolyticus, A. georgiae*, *A. naeslundii*, and *A. oris* were the other species identified in the remaining 25% of the cases (Table 1). Actinomycosis is usually considered a polymicrobial infection involving anaerobic and aerobic bacteria. Co-isolates depend on the site or source of infection. In 35 cases (30%), Co-isolates were identified along with actinomycosis. Most isolates were related to fusobacterium species (n = 14), followed by Streptococcus species (Additional file 1 s5, Table 1).

**Table 4:** Radiological and laboratory diagnosis of CNS actinomycosis

CNS imaging/diagnostic procedures	N (%)
Brain Abscess	65/118 (55.1)
Single	49/65 (75.4)
Multiple	16/65 (24.6)
**Abscess location**^**a**^	
Frontal	32 (39.5)
Parietal	23 (28.4)
Temporal	11 (13.6)
Occipital	9 (11.1)
Cerebellar	6 (7.4)
Leptomeningeal enhancement	26 (22)
Subdural empyema	17 (14.4)
Cerebral venous thrombosis/septic emboli/infarct	20 (16.9)
Spinal cord involvement (abscess)^b^	19 (16.1)
Hydrocephalus/Ventriculitis	5 (4.2)
**Culture positive**	**63 (53.4)**
**Source**	
Brain abscess aspiration	36/65 (55.4)
CSF culture	11/65 (16.9)
Blood culture	4 /65 (6.1)
Other tissue^c^	4/65 (6.1)
Culture site not specified	8/65 (12.2)
**CSF analysis**	
Neutrophilic pleocytosis	13/19 (68.4)
Median total leukocyte counts, (IQR)	548.5 (133–1380)
Median CSF protein, mg/dl, (IQR)	112 (68.3–214.3)
Median CSF Glucose, mg/dl, (IQR)	40 (26–54)

^a^Some of the patients had multiple abscess, with involving more than one lobe**,**

^b^5 patients = cervical, 5 patients = lumber, 3 patients = thoracic, 3 patients = thoraco-lumbar, 2 patients = lumbo-sacral, and 1 patient with sacral abscess

^c^3 patients with lung abscess, 1 patients with chest wall abscess, IQR = inter quartile range

Among the neuroimaging findings, focal space-occupying lesions (brain abscess) were the most frequent (55%). The majority of these lesions were single abscesses (Table 4). The frontal and parietal lobes were the most common site of brain abscesses (Table 4). Leptomeningeal enhancement, subdural empyema, and ventriculitis were the other neuroimaging findings in CNS actinomycosis (Table 4). Spinal cord involvement in the form of intradural abscess is also found in 19 cases, and the cervical and lumbar region were the site affected most frequently (Table 4). On neuroimaging, brain abscess incidence was significantly higher in disseminated cases compared to isolated CNS disease (73% vs 52%, p = 0.034).

### Treatment and outcome

Actinomycosis is a chronic suppurative indolent disease that usually requires prolonged antimicrobial therapy to effectively cure the infection and prevent relapse. In this review, surgical intervention (abscess drainage or tissue debridement) was performed in 79 (67%) patients. The mean antimicrobial duration was 160 days for treating CNS actinomycosis. On a few occasions, therapy was continued beyond one year (up to 720 days).

The mean duration of antimicrobials was 150 days in isolated CNS cases compared to 200 days in disseminated actinomycosis. Single antibiotic use was preferred in 58.7% of cases; however, combination antimicrobials were also used in 41.3% of patients (Table 5). Of note, combination antimicrobial' use was significantly higher in disseminated cases than in isolated CNS disease (65% vs 37%, p = 0.001). There was a wide heterogenicity in antibiotic preference in all cases. Penicillin was the most frequently used antimicrobial, followed by ceftriaxone and ampicillin (47%, 29% and 11%, respectively). Among oral antimicrobials (patients who were discharged after initial iv. Antibiotics), amoxicillin, cotrimoxazole, and doxycycline were preferred drugs. Some recent reports also describe the use of meropenem in CNS actinomycoses [23, 24].

**Table 5:** Treatment and outcome in patients with CNS actinomycosis

Variables	N (%)
Mean antimicrobial duration (days)	160
**Antibiotic treatment**	
Single	61/104 (58.7)
Combination antimicrobials	43/104 (41.3)
Not specified	14
**Type of antimicrobial therapy**	
1. Penicillin based regimen	47
Penicillin alone	20
Combination with other antibiotics^a^	27
2. Ceftriaxone based regimen	29
Ceftriaxone alone	3
Combination with other antibiotics^b^	26
3. Ampicillin based regimen	11
Ampicilllin. Alone	4
Combination of other antibiotics^c^	7
4. Meropenem	5
**Outcome**	
Patient died	13/118 (11)
Survived with sequelae	17/77 (22)
Survived without sequelae	60/77 (78)

^a^other antibiotics = cotrimoxazole/amoxicillin/doxycycline

^b^other antibiotics = ciprofloxacin/amoxicillin/clindamycin/erythromycin

^c^other antibiotics = doxycycline/clindamycin/amoxicillin,

The exact antimicrobial regimen and duration of therapy are not well defined in actinomycosis. We also analyzed the role of combination therapy, type of antibiotics and duration of treatment on clinical outcomes. Survival rate was similar in patients who received combination antibiotic compared to those treated with single drug (Table 6). However, neurological sequelae were significantly less in patients treated with combination therapy (Table 6). Compared to the penicillin-based regimen, ceftriaxone-based treatment was associated with lesser residual deficits though the mortality rate did not differ (Table 6). Those who received more extended treatment (> three months) had significantly fewer neurological sequelae (Table 6). We also analyzed the impact of polymicrobial infections (a common occurrence in actinomycosis) on the clinical outcome of CNS actinomycosis. We did not find any difference in outcomes (mortality and relapse) in polymicrobial infection compared to monomicrobial (actinomycosis alone) (Additional file 1: s6, Table 2). These patients (with polymicrobial disease) had received more extended antibiotic therapy (202 days vs 136 days, *p *value 0.02) and a higher proportion of combination antibiotics. The clinical significance of polymicrobial infection and the need for antibiotics for other co-isolates is still debatable, it should be decided on case-to-case basis.

**Table 6:** The association of various antibiotic regimen and treatment duration with outcome in CNS actinomycosis

Variables	Single antibiotic	Combination antibiotic regimen	*p *value
Mortality	3/43 (6.9%)	5/61 (8.2%)	0.56
Neurological sequelae	10/32 (31.2%)	5/43 (11.6%)	0.03
	Penicillin based regimen	Ceftriaxone based regimen	
Mortality	2/45 (4.4%)	1/27 (3.7%)	0.60
Neurological sequelae	6/33 (18.2%)	3/27 (11.1%)	0.34
	Antibiotic duration ≤ 3 months	Antibiotic duration > 3 months	
Neurological sequelae	11/38 (28.9%)	5/43 (11.6%)	0.04

The overall mortality rate was 11% (n = 13) in all cases reported with CNS actinomycosis. We have also tried to look for residual neurological deficits in patients whose long-term follow-up (at least one year after treatment initiation) was available. In this review, 22% of patients with CNS actinomycosis had residual neurological sequelae at the end of 12 months (Table 7). We further categorized all patients into survived and non-survived groups to find out various predictors of mortality. The comparison of both groups for different predictors of mortality is shown in Table 7. Advanced age (≥ 65 years) and surgical intervention were found to be statistically significant determinant factors for the outcome (Table 7). After adjusting for other factors (age ≥ 65, immunodeficiency state), only surgical intervention was found to be an independent predictor of survival. The patients who underwent surgery and antimicrobial therapy were less likely to die compared to those treated with antimicrobial therapy alone (adjusted OR 0.14, 95% CI 0.04–0.28, *p *value 0.039).

**Table 7:** Clinical factors associated with poor treatment outcomes in patients with CNS actinomycosis

Variable	Univariate analysis		Multivariate analysis	
	*P* value	Crude OR (95% CI)	*P* value	Crude OR (95% CI)
Age ≥ 65 (years)	0.041	3.1, (1.2–11.69)	0.414	1.6, (0.32–4.8)
Comorbidities	0.368	1.2, (0.38–4.0)		
Immunodeficient state	0.10	2.5, (0.61–10.75)	0.293	1.8, (0.76–5.8)
Fever	0.179	0.54, (0.15–1.89)		
Multiple brain abscess	0.401	1.18, (0.23–5.90)		
Disseminated disease	0.153	2.2, (0.56–7.2)		
Combination antibiotics	0.155	0.48, (0.12–1.87)		
Surgery	< 0.001	0.028, (0.003–0.23)	0.039	0.14, (0.04–0.28)

OR = Odds ratio, CI = Confidence interval, p value < 0.05 considered significant

## Discussion

Actinomycosis is a rare, invasive bacterial disease characterized by chronic granulomatous infection and pus discharging sinuses. CNS involvement is uncommon in actinomycosis, but can cause significant morbidity and mortality. Moreover, data are scarce due to the disease's rarity and the low index of suspicion. Most literature is available in the form of anecdotal data with a lack of more extensive studies and treatment guidelines. The treatment decision for CNS disease depends on the literature predominantly focused on cervicofacial and pulmonary actinomycosis, which have a different clinical spectrum from CNS disease. This review of published cases of CNS actinomycosis was conducted to provide insight into the aforementioned factors.

Actinomycosis is usually considered a disease with low virulence. CNS spread primarily depends on mucosal breach and contiguous spread from cervicofacial areas, skull osteomyelitis and less commonly from hematogenous spread from distant foci (lungs, abdomen, pelvis) [25]. Trauma, surgery, dental extraction, and ear infections are the usual trigger for invasive CNS actinomycosis. The incidence of CNS disease is 1–2% in all disseminated cases of actinomycosis [26, 27]. In this review, disseminated infection was found in 20% of the patients, which was probably due to hematogenous spread. The primary source of actinomycosis was the lungs in 10% of the cases. A previous review by Smego et al. described the relatively high proportion of pulmonary actinomycosis (27%) in disseminated cases [14]. In their report, cyanotic heart disease (CHD) was also described as a risk factor for actinomycosis brain abscess. However, we did not find any patients with CHD. The common imitators of actinomycosis are Nocardia and tuberculosis, which typically develop in immunodeficient patients, CNS actinomycosis is not usually associated with immunodeficiency. Only 12% of the cases were found immunodeficient. A large multicenter study reported the low prevalence of actinomycosis in renal transplant recipients (0.02%), and none of these patients had CNS disease [28]. Smego et al. also reported that only 7% of patients were immunocompromised [14]. This also emphasizes the need for a high index of suspicion of CNS actinomycosis in immunocompetent patients. The clinical presentation of CNS actinomycosis depends on the site and extent of the lesion. Focal neurological deficits and headache are the common presentations of CNS disease, which makes it indistinguishable from other chronic CNS infections (e.g., nocardiosis and neuroaspergillosis). The indolent nature and lack of fever at presentation prompt an erroneous diagnosis of malignancy in some cases of actinomycosis. Meningitis is an uncommon presentation in CNS actinomycosis. In this review, the incidence of meningitis was 21% which was significantly lower compared to the previous review (46%) [14]. Moreover, isolated meningitis was seen in only 11% of the cases. A high proportion of meningitis in previous studies might be related to delayed presentation, leading to the rupture of foci into the subarachnoid space, producing meningeal signs and CSF abnormalities.

The diagnosis of actinomycosis is based on a constellation of histopathological features and microbiological isolation of the organism [6, 29]. However, failure to isolate the organism from culture does not exclude the diagnosis. Many of these patients had suppurative disease and received multiple antibiotics that could preclude culture positivity. In this review, nearly half of the patients were culture-negative. Actinomycosis is a fastidious organism which requires a prolonged incubation period (14–21 days) in an anaerobic environment [6]. Suppression by concomitant pathogens and inadequate culture media are the other reasons for culture negativity. *Actinomycosis israelii* is the most common causative agent for CNS disease. We found *A. meyeri* and *A. viscosus* as emerging species in the last two decades causing CNS infections, which were not identified in a previous review published in 1987 [14]. Both these species are known to cause invasive actinomycosis and bacteraemia (3 out of 4 cases in this review). We found a low CSF culture positivity, which may be explained due to the low prevalence of meningitis and the inadequate samples sent for culture. CSF cytology showed predominantly neutrophilic leucocytosis, in contrast to the lymphocytic predominance seen in other mimickers like neuroaspergillosis [30]. Another characteristic of actinomycosis infection is the concomitant microorganism which grew in the culture. This polymicrobial infection acts in synergy, and the other pathogens decrease the oxygen tension, thus enhancing the growth of actinomycosis. In addition, the host defence mechanism is also suppressed in this setting [31, 32].

The demonstration of characteristic sulfur granules also supports the diagnosis of actinomycosis though it can also be seen in nocardiosis and botryomycosis [33]. Sulfur granules have a tiny, yellowish tinge composed of filamentous bacteria, and at the periphery, there are eosinophilic filaments with club ends [5, 33]. CNS cases usually have delayed presentation; by that time, the lesion may have extensive fibrosis, which can impair the identification of sulfur granules. Recent reports highlight the role of targeted histological evaluation in increasing the detection of actinomycosis [34]. The advent of molecular techniques (PCR, 16S rRNA gene sequencing) allows the rapid identification of actinomycosis species. Recent reports have also shown promising results of MALDI-TOF comparable to 16S rRNA sequencing [35]. Neuroimaging in CNS actinomycosis shows brain abscess in more than 50% of the cases [14], similar to nocardiosis and neuroaspergillosis [30, 36]. The high predilection of the temporal lobe was reported in the previous systematic review [14]. However, in this review, the frontal and frontal-parietal lobes were the most common area affected.

In this study, the mortality rate was 11% in all treated cases, which is lower compared to previous review (28%), [14]. In last few decades, prompt diagnosis, availability of antibiotics and early aggressive surgery could be the potential factors contributing to low mortality. Medical management of CNS actinomycosis includes a prolonged high dose of antimicrobials. Determining the optimal duration and use of combination antibiotics remains elusive in CNS disease. The decision should be individualized based on the severity of the disease, clinical response and immune status of the patients [1, 6]. We recommend at least 3–6 months of antibiotics in CNS actinomycosis, which can be extended if an adequate clinical response is not achieved or source control (surgery) is not possible. Antibiotic susceptibility is usually not essential, with most isolates showing a good sensitivity to beta-lactam antibiotics (penicillin, amoxicillin, ceftriaxone, meropenem, piperacillin-tazobactam), [1]. High-dose penicillin is the most widely used therapy for CNS disease [14]. However, we found the emerging use of ceftriaxone, ampicillin and meropenem. Amoxicillin, clindamycin, doxycycline and cotrimoxazole are good options for maintenance therapy. Of note, the clinician should be aware of a few actinomycosis species (*A. turicensis* and *A. europaeus*) that showed resistance to clindamycin, macrolides and quinolones [37, 38]. Metronidazole and aminoglycosides usually do not have any activity against actinomycosis and should be avoided [39]. Though combination therapy was the preferred approach based on this review, it did not improve the outcome of CNS actinomycosis. Further studies should focus on this elusive question. Surgical intervention (debridement of necrotic tissue, abscess drainage) becomes vital in CNS disease. In this review, early surgery was found to be an independent factor in improving the outcomes. Thus, it should not be reserved only for cases with a poor response to antibiotics. Whether it will shorten the hospital stay and antibiotic duration is a matter of further studies.

This systematic review has a few limitations; firstly, case reports are inherently biased; a heterogeneous population makes the outcome and risk factor analysis difficult. Data regarding the antimicrobial susceptibility reports are not available. Many cases were excluded due to a lack of information regarding treatment and follow-up.

## Conclusions

Despite the indolent nature of the disease, CNS actinomycosis carries significant mortality. Early suspicion and differentiation from tuberculosis, nocardiosis and malignancy is vital to improve outcomes. The diagnostic utility of molecular methods could be helpful in such settings. We emphasize early aggressive surgery and prolonged antibiotics in CNS actinomycosis to improve clinical outcomes. The use of combination antibiotics is debatable and requires further evidence in terms of prospective studies.

## Supplementary Information

**Additional file 1: Supplemental 1.** PRISMA checklist.** Supplemental 2.** Search Strategy Report.** Supplemental 3:** JBI Critical Appraisal Checklist for Case Reports.** Supplemental 4.** All references included in this review.** Supplemental 5. Table S1.** Other Microorganisms isolated in CNS Actinomycosis.** Supplemental 6. Table S2.** The impact of polymicrobial infections on clinical outcome in CNS actinomycosis.

## Data Availability

The datasets used and/or analysed during the current study available from the corresponding author on reasonable request.

## Abbreviations

PRISMA: Preferred reporting items for systematic reviews and meta-Analyses
JBI: Joanna Briggs Institute
CIs: Confidence intervals
MALDI-TOF: Matrix-Assisted Laser Desorption/Ionization-Time of Flight
CHD: Cyanotic heart disease
CSF: Cerebrospinal fluid
