# Supplementary material for: The epidemiology, clinical presentation and treatment outcomes in CNS actinomycosis: a systematic review of reported cases
Source: Orphanet J Rare Dis. 2023 Jun 2;18:133. doi: 10.1186/s13023-023-02744-z (PMC10239160; doi:10.1186/s13023-023-02744-z)
Supplement: Supplementary file 1 — Additional file 1: Supplemental 1. PRISMA checklist. Supplemental 2. Search Strategy Report. Supplemental 3: JBI Critical Appraisal Checklist for Case Reports. Supplemental 4. All references included in this review. Supplemental 5. Table S1. Other Microorganisms isolated in CNS Actinomycosis. Supplemental 6. Table S2. The impact of polymicrobial infections on clinical outcome in CNS actinomycosis. [file 13023_2023_2744_MOESM1_ESM.docx]

**Supplemental files**

**Supplemental 1: PRISMA checklist**

| **Section/topic** | **#** | **PRISMA Checklist item** | **Reported on page #** |
| --- | --- | --- | --- |
| **TITLE** | | |  |
| Title | 1 | Identify the report as a systematic review, meta-analysis, or both. | 1 |
| **ABSTRACT** | | |  |
| Structured summary | 2 | Provide a structured summary including, as applicable: background; objectives; data sources; study eligibility criteria, participants, and interventions; study appraisal and synthesis methods; results; limitations; conclusions and implications of key findings; systematic review registration number. | 1 |
| **INTRODUCTION** | | |  |
| Rationale | 3 | Describe the rationale for the review in the context of what is already known. | 2 |
| Objectives | 4 | Provide an explicit statement of questions being addressed with reference to participants, interventions, comparisons, outcomes, and study design (PICOS). | 2 |
| **METHODS** | | |  |
| Protocol and registration | 5 | Indicate if a review protocol exists, if and where it can be accessed (e.g., Web address), and, if available, provide registration information including registration number. | 3 |
| Eligibility criteria | 6 | Specify study characteristics (e.g., PICOS, length of follow-up) and report characteristics (e.g., years considered, language, publication status) used as criteria for eligibility, giving rationale. | 3-4 |
| Information sources | 7 | Describe all information sources (e.g., databases with dates of coverage, contact with study authors to identify additional studies) in the search and date last searched. | Supplement 2,4 |
| Search | 8 | Present full electronic search strategy for at least one database, including any limits used, such that it could be repeated. | Supplement 2 |
| Study selection | 9 | State the process for selecting studies (i.e., screening, eligibility, included in systematic review, and, if applicable, included in the meta-analysis). | 4 |
| Data collection process | 10 | Describe method of data extraction from reports (e.g., piloted forms, independently, in duplicate) and any processes for obtaining and confirming data from investigators. | 4 |
| Data items | 11 | List and define all variables for which data were sought (e.g., PICOS, funding sources) and any assumptions and simplifications made. | N/A |
| Risk of bias in individual studies | 12 | Describe methods used for assessing risk of bias of individual studies (including specification of whether this was done at the study or outcome level), and how this information is to be used in any data synthesis. | 4,5 |
| Summary measures | 13 | State the principal summary measures (e.g., risk ratio, difference in means). | N/A |
| Synthesis of results | 14 | Describe the methods of handling data and combining results of studies, if done, including measures of consistency (e.g., I^2^) for each meta-analysis. | N/A |
| **RESULTS** | | |  |
| Study selection | 17 | Give numbers of studies screened, assessed for eligibility, and included in the review, with reasons for exclusions at each stage, ideally with a flow diagram. | Figure 1 |
| Study characteristics | 18 | For each study, present characteristics for which data were extracted (e.g., study size, PICOS, follow-up period) and provide the citations. | 4 |
| Risk of bias within studies | 19 | Present data on risk of bias of each study and, if available, any outcome level assessment (see item 12). | Supplement 3 |
| Results of individual studies | 20 | For all outcomes considered (benefits or harms), present, for each study: (a) simple summary data for each intervention group (b) effect estimates and confidence intervals, ideally with a forest plot. | Table 1-5 |
| Synthesis of results | 21 | Present results of each meta-analysis done, including confidence intervals and measures of consistency. | N/A |
| Risk of bias across studies | 22 | Present results of any assessment of risk of bias across studies (see Item 15). | N/A |
| Additional analysis | 23 | Give results of additional analyses, if done (e.g., sensitivity or subgroup analyses, meta-regression [see Item 16]). | N/A |
| **DISCUSSION** | | |  |
| Summary of evidence | 24 | Summarize the main findings including the strength of evidence for each main outcome; consider their relevance to key groups (e.g., healthcare providers, users, and policy makers). | 9-12 |
| Limitations | 25 | Discuss limitations at study and outcome level (e.g., risk of bias), and at review-level (e.g., incomplete retrieval of identified research, reporting bias). | 13 |
| Conclusions | 26 | Provide a general interpretation of the results in the context of other evidence, and implications for future research. | 13 |
| **FUNDING** | | |  |
| Funding | 27 | Describe sources of funding for the systematic review and other support (e.g., supply of data); role of funders for the systematic review. | 13 |

**Supplemental 2**

Search Strategy Report

PubMed

PubMed (684 Documents)

"CNS"[All Fields] OR intracranial[All Fields] OR ("brain abscess"[MeSH Terms] OR ("brain"[All Fields] AND "abscess"[All Fields]) OR "brain abscess"[All Fields]) OR ("meningitis"[MeSH Terms] OR "meningitis"[All Fields]) OR spinal[All Fields] AND ("actinomycosis"[MeSH Terms] OR "actinomycosis"[All Fields]) AND ("1988/01/01"[PubDate] : "2022/12/31"[PubDate])

Google Scholar (262 documents)

“CNS" or “intracranial" or "brain abscess" or “meningitis" OR “spinal” OR “epidural abscess” and “actinomycosis”

Scopus (345 documents)

(TITLE-ABS-KEY ( CNS AND infection ) OR TITLE-ABS-KEY ( intracranial AND infection ) OR TITLE-ABS-KEY ( brain AND abscess ) OR TITLE-ABS-KEY ( meningitis ) OR TITLE-ABS-KEY ( spinal ) OR TITLE-ABS-KEY ( epidural AND abscess ) AND TITLE-ABS-KEY ( actinomycosis ) )

**Supplemental 3: Quality assessment**

Table1: JBI Critical Appraisal Checklist for Case Reports

| Study reference | Were patient’s demographic characteristics clearly described? | Was the patient’s history clearly described and presented as a timeline?? | Was the current clinical condition of the patient on presentation clearly described? | Were diagnostic tests or methods and the results clearly described? | Was the intervention(s) or treatment procedure(s) clearly described? | Was the post-intervention clinical condition clearly described? | Were adverse events (harms) or unanticipated events identified and described?? | Does the case report provide takeaway lessons? | Risk of bias^a^ |
| --- | --- | --- | --- | --- | --- | --- | --- | --- | --- |
| Smego et al., 1987 | yes | yes | yes | yes | yes | yes | yes | yes | low |
| Hotte et al., 2019 | yes | yes | no | no | yes | yes | no | yes | moderate |
| Hwang et al., 2018 | yes | yes | no | yes | yes | yes | no | yes | moderate |
| Wang et al.,2020 | yes | yes | yes | yes | yes | yes | yes | yes | low |
| Ham et al., 2011 | yes | yes | yes | yes | yes | yes | no | yes | low |
| Chicoine et al., 2021 | yes | yes | yes | yes | yes | yes | yes | yes | low |
| Rahiminejad et al.,2016 | yes | yes | yes | yes | yes | yes | yes | yes | low |
| Bonnefond et al., 2016 | yes | yes | yes | yes | yes | yes | yes | yes | low |
| Sah et al., 2020 | yes | yes | no | yes | yes | yes | no | yes | low |
| Narayan et al., 2009 | yes | yes | yes | no | yes | yes | no | yes | low |
| Hotte et al., 2019 | yes | yes | yes | yes | yes | yes | no | yes | low |
| Hwang et al., 2018 | yes | yes | no | yes | yes | yes | yes | yes | low |
| Wang et al., 2020 | yes | yes | no | yes | yes | yes | yes | yes | low |
| Ham et al., 2011 | yes | yes | no | yes | yes | yes | yes | yes | low |
| Ismail et al., 2015 | yes | yes | yes | yes | yes | yes | yes | yes | low |
| Kocsis et al., 2018 | yes | yes | yes | yes | yes | no | yes | yes | low |
| Mohamad et al., 2021 | yes | yes | yes | yes | no | yes | yes | yes | low |
| Abo-zed et al., 2021 | yes | yes | no | no | yes | yes | no | yes | moderate |
| Deora et al., 2018 | yes | yes | no | no | yes | yes | no | yes | moderate |
| Verma et al., 2008 | yes | yes | no | no | yes | yes | no | yes | moderate |
| Guillamet et al., 2017 | yes | yes | no | no | yes | yes | no | yes | moderate |
| Pareira et al., 2022 | yes | yes | no | no | yes | yes | no | yes | moderate |
| Funakoshi et al., 2020 | yes | yes | no | no | no | yes | no | yes | high |
| Narayanan et al., 2012 | yes | yes | no | no | yes | yes | no | yes | moderate |
| Chen et al., 2013 | yes | yes | no | yes | yes | no | yes | yes | moderate |
| Jain et al., 2021 | yes | yes | no | no | yes | yes | yes | yes | moderate |
| Miller et al., 2014 | yes | yes | yes | yes | yes | yes | yes | yes | low |
| Hadgaonkar et al., 2021 | yes | yes | no | yes | yes | yes | no | yes | low |
| stimac et al., 2020 | yes | yes | no | no | yes | yes | no | yes | moderate |
| Yang et al., 2019 | yes | yes | no | yes | yes | yes | yes | yes | low |
| Clancy et al., 2015 | yes | yes | yes | yes | yes | yes | yes | yes | low |
| Anderson et al., 2014 | yes | yes | no | yes | yes | yes | no | yes | moderate |
| Simpson et al., 1996 | yes | yes | yes | yes | yes | yes | yes | yes | low |
| Kumar et al., 2014 | yes | yes | no | yes | no | yes | no | yes | moderate |
| Perez et al., 2021 | yes | yes | yes | yes | yes | yes | no | yes | low |
| Vargas et al., 2020 | yes | yes | yes | yes | yes | no | yes | yes | low |
| Kapmaz et al., 2014 | yes | yes | no | yes | yes | yes | no | yes | low |
| Cano et al., 2020 | yes | yes | no | yes | yes | yes | no | yes | low |
| Park et al., 2011 | yes | yes | no | yes | yes | yes | no | yes | low |
| Bellingan et al., 1990 | yes | yes | yes | yes | yes | yes | no | yes | low |
| Yong na et al., 2013 | yes | yes | yes | yes | yes | yes | yes | yes | low |
| Park et al., 2014 | yes | yes | no | no | no | yes | yes | yes | moderate |
| Funaki et al., 1998 | yes | yes | yes | yes | yes | yes | no | yes | low |
| Honda et al., 2008 | yes | yes | yes | yes | yes | yes | yes | yes | low |
| Kim et al., 2017 | yes | yes | no | no | yes | yes | no | yes | moderate |
| Fadda et al., 2014 | yes | yes | no | yes | no | yes | no | yes | moderate |
| Hagiya et al., 2014 | yes | yes | no | no | no | yes | no | yes | high |
| Patil et al., 2014 | yes | yes | yes | yes | yes | no | yes | yes | low |
| Watkins et al., 2008 | yes | yes | yes | yes | yes | yes | yes | yes | low |
| Holland et al., 1998 | yes | yes | yes | yes | yes | yes | no | yes | low |
| Shen et al., 2018 | yes | yes | yes | yes | yes | yes | yes | yes | low |
| Kobayashi et al., 2020 | yes | yes | yes | yes | yes | yes | yes | yes | low |
| Habib et al., 2018 | yes | yes | yes | yes | yes | yes | yes | yes | low |
| Grach et al., 2020 | yes | yes | yes | yes | yes | yes | yes | yes | low |
| Hagiya et al., 2014 | yes | yes | yes | yes | yes | no | yes | yes | low |
| Patil et al., 2014 | yes | yes | yes | yes | yes | yes | yes | yes | low |
| Watkins et al., 2008 | yes | yes | yes | yes | yes | yes | yes | yes | low |
| Holland et al., 1998 | yes | yes | yes | yes | no | yes | yes | yes | low |
| Shen et al., 2018 | yes | yes | yes | yes | yes | yes | yes | yes | low |
| Kobayashi et al., 2020 | yes | yes | yes | yes | yes | yes | yes | yes | low |
| Habib et al., 2018 | yes | yes | yes | yes | yes | yes | yes | yes | low |
| Vikas et al., 2014 | yes | yes | yes | yes | yes | yes | yes | yes | low |
| Puzzulli et al., 1998 | yes | yes | no | yes | yes | yes | no | yes | moderate |
| King et al., 1998 | yes | yes | yes | yes | yes | yes | yes | yes | low |
| Ryu et al., 2020 | yes | yes | yes | yes | yes | no | no | yes | moderate |
| Gaini et al., 2006 | yes | yes | yes | yes | yes | no | yes | yes | low |
| Olah et al., 2004 | yes | yes | yes | yes | no | yes | yes | yes | low |
| sharma et al., 1990 | yes | yes | yes | yes | no | yes | no | yes | moderate |
| Ravindra et al., 2018 | yes | no | yes | yes | no | yes | no | no | high |
| Ravindra et al., 2018 | yes | yes | yes | yes | no | yes | no | yes | moderate |
| Ravindra et al., 2018 | yes | yes | yes | yes | no | yes | yes | yes | low |
| Ravindra et al., 2018 | yes | yes | yes | no | yes | yes | yes | yes | low |
| Ravindra et al., 2018 | yes | yes | yes | yes | yes | yes | no | yes | low |
| Ravindra et al., 2018 | yes | yes | yes | yes | yes | yes | no | yes | low |
| Ravindra et al., 2018 | yes | yes | yes | yes | yes | no | no | yes | low |
| Akhaddar et al., 2010 | yes | yes | no | no | yes | no | no | yes | high |
| Akhaddar et al., 2010 | yes | yes | yes | yes | yes | yes | no | yes | low |
| Akhaddar et al., 2010 | yes | yes | yes | yes | yes | yes | no | yes | low |
| Akhaddar et al., 2010 | yes | yes | no | yes | yes | yes | no | yes | low |
| Akhaddar et al., 2010 | yes | yes | yes | yes | yes | yes | no | yes | low |
| Jamjoom et al 1994 | yes | yes | yes | yes | yes | yes | no | yes | low |
| Jamjoom et al., 1994 | yes | yes | no | yes | no | yes | no | no | high |
| Jamjoom et al., 1994 | yes | yes | yes | yes | yes | yes | no | yes | low |
| Fabbrii et al., 2014 | yes | yes | yes | yes | yes | yes | yes | yes | low |
| Budenz et al., 2010 | yes | yes | no | yes | yes | no | yes | yes | low |
| Tsai et al., 2001 | yes | yes | yes | yes | yes | yes | yes | yes | low |
| Lad et al., 1991 | yes | yes | yes | yes | yes | yes | no | yes | low |
| Lad et al., 1991 | yes | yes | yes | yes | yes | yes | no | no | moderate |
| Lad et al., 1991 | yes | no | yes | yes | yes | yes | no | yes | low |
| Navas et al., 1994 | yes | yes | yes | yes | yes | yes | yes | yes | low |
| Lagunes et al., 2017 | yes | yes | yes | yes | no | yes | yes | yes | low |
| Nugent et al., 2010 | yes | yes | yes | yes | no | yes | yes | yes | low |
| Mohindra et al., 2012 | yes | yes | yes | yes | yes | yes | yes | yes | low |
| Mohindra et al., 2012 | yes | yes | yes | yes | yes | yes | yes | yes | low |
| Mishra et al., 2019 | yes | yes | no | yes | no | no | no | yes | high |
| Ghobrial et al., 2016 | yes | yes | no | yes | no | yes | no | yes | moderate |
| Opsomer et al., 2019 | yes | yes | yes | yes | yes | yes | yes | yes | low |
| Hernandez et al., 1999 | yes | yes | yes | yes | yes | yes | yes | yes | low |
| Haggerty et al., 2012 | yes | yes | yes | yes | yes | yes | yes | yes | low |
| Koda et al., 2003 | yes | yes | no | no | no | yes | no | yes | high |
| Rahiminejad et al., 2016 | yes | yes | yes | yes | yes | yes | yes | yes | low |
| Imamura et al., 2011 | yes | yes | yes | yes | no | yes | yes | yes | low |
| Chotmongkol et al., 2002 | yes | yes | yes | yes | no | yes | yes | yes | low |
| Adeyemi et al., 2008 | yes | yes | yes | yes | yes | yes | yes | yes | low |
| Saleem et al., 2017 | yes | yes | yes | yes | yes | yes | yes | yes | low |
| Ohta et al., 2002 | yes | yes | yes | yes | yes | no | yes | yes | low |
| Limaye et al., 2021 | yes | yes | yes | yes | yes | yes | yes | yes | low |
| Ushikoshi et al., 1998 | yes | yes | yes | yes | yes | yes | yes | yes | low |
| Ipek et al., 2017 | yes | yes | yes | yes | yes | yes | no | yes | low |
| Khare et al., 2019 | yes | yes | no | yes | yes | yes | no | yes | moderate |
| Trisha et al., 2010 | yes | yes | no | yes | no | yes | no | no | high |
| Takahashi et al., 2020 | yes | yes | yes | yes | yes | yes | yes | yes | low |
| Lubomski et al., 2018 | yes | yes | yes | yes | yes | yes | yes | yes | low |
| Dua et al., 2010 | yes | yes | yes | no | no | yes | yes | yes | moderate |
| Dua et al., 2010 | yes | yes | yes | yes | no | yes | yes | yes | low |
| Bouziri et al., 2011 | yes | yes | no | yes | yes | yes | yes | yes | low |
| Kumar et al., 2019 | yes | yes | yes | yes | yes | yes | yes | yes | low |
| Douleh et al., 2016 | yes | yes | yes | yes | yes | yes | yes | yes | low |
| Hall et al., 1994 | yes | yes | no | no | yes | no | no | yes | high |
| Adler et al., 1992 | yes | no | yes | yes | yes | no | no | yes | moderate |
| Wang et al., 2017 | yes | yes | yes | yes | yes | yes | yes | yes | low |
| Harshith et al., 2019 | yes | yes | yes | yes | yes | yes | yes | yes | low |

^a^ Risk based on eight questions (Low risk = ≥ 7 yes; Moderate risk = 5-6 yes; High risk = ≤ 4 yes).

**Supplemental-4 (All references included in this review)**

1. Smego RA Jr. Actinomycosis of the central nervous system. Rev Infect Dis. 1987 Sep-Oct;9(5):855-65.
2. Hötte GJ, Koudstaal MJ, Verdijk RM, Titulaer MJ, Claes JFHM, Strabbing EM, van der Lugt A, Paridaens D. Intracranial actinomycosis of odontogenic origin masquerading as auto-immune orbital myositis: a fatal case and review of the literature. BMC Infect Dis. 2019 Sep 2;19(1):763.
3. Hwang CS, Lee H, Hong MP, Kim JH, Kim KS. Brain abscess caused by chronic invasive actinomycosis in the nasopharynx: A case report and literature review. Medicine (Baltimore). 2018 Apr;97(16):e0406.
4. Wang Y, Ren X, Shen D, Mao C, Wang H, Peng B, Gao J, Cui L. Spinal Intrathecal Actinomycosis Causes Multisegmental Root Failure: A Case Report. Front Neurol. 2020 Jun 30;11:621. doi: 10.3389/fneur.2020.00621. PMID: 32714273; PMCID: PMC7344188.
5. Ham HY, Jung S, Jung TY, Heo SH. Cerebral actinomycosis : unusual clinical and radiological findings of an abscess. J Korean Neurosurg Soc. 2011 Aug;50(2):147-50. doi: 10.3340/jkns.2011.50.2.147. Epub 2011 Aug 31. PMID: 22053238; PMCID: PMC3206280.
6. Chicoine NH, Griffith-Linsley J, Goh J, Manaloor JJ, Raskin JS. Giant Actinomyces brain abscess in an immunocompetent child: A management strategy. Surg Neurol Int. 2021 Jul 6;12:325. doi: 10.25259/SNI_164_2021. PMID: 34345466; PMCID: PMC8326064.
7. Rahiminejad M, Hasegawa H, Papadopoulos M, MacKinnon A. Actinomycotic brain abscess. BJR Case Rep. 2016 Nov 2;2(4):20150370. doi: 10.1259/bjrcr.20150370. PMID: 30460021; PMCID: PMC6243313.
8. Bonnefond S, Catroux M, Melenotte C, Karkowski L, Rolland L, Trouillier S, Raffray L. Clinical features of actinomycosis: A retrospective, multicenter study of 28 cases of miscellaneous presentations. Medicine (Baltimore). 2016 Jun;95(24):e3923.
9. Sah R, Nepal G, Sah S, Singla S, Upadhyay P, Rabaan AA, Dhama K, Rodriguez-Morales AJ, Ghimire R. A rare case of brain abscess caused by Actinomyces meyeri. BMC Infect Dis. 2020 May 27;20(1):378.
10. Narayan SK, Swaroop A, Jayanthi S. Chronic epidural intracranial actinomycosis: A rare case. Ann Indian Acad Neurol. 2009 Jul;12(3):195-6. doi: 10.4103/0972-2327.56324.
11. Ismail NJ, Bot GM, Sahabi S, Aliu S, Usman B, Shilong DJ, Obande JO, Shehu BB. Subdural actinomycoma presenting as recurrent chronic subdural hematoma. Asian J Neurosurg. 2015 Apr-Jun;10(2):129-31. doi: 10.4103/1793-5482.145051.
12. Kocsis B, Tiszlavicz Z, Jakab G, Brassay R, Orbán M, Sárkány Á, Szabó D. Case report of Actinomyces turicensis meningitis as a complication of purulent mastoiditis. BMC Infect Dis. 2018 Dec 20;18(1):686. doi: 10.1186/s12879-018-3610-y.
13. Mohamad AR, Koleri J, Hussain HMS, Al Soub H, Al Maslamani M. Recurrent skull vault actinomycosis: A case report and review of literature. IDCases. 2021 Jul 3;25:e01215. doi: 10.1016/j.idcr.2021.e01215.
14. Abo-Zed A, Yassin M, Phan T. A rare case of polymicrobial brain abscess involving *Actinomyces*. Radiol Case Rep. 2021 Mar 4;16(5):1123-1126. doi: 10.1016/j.radcr.2021.02.042.
15. Deora H, Beniwal M, Rao S, Rao KVLN, Vikas V, Somanna S. Wolf in Sheep's clothing: Intracranial actinomycosis masquerading as *en-plaque* meningioma. Surg Neurol Int. 2018 Feb 14;9:39. doi: 10.4103/sni.sni_445_17.
16. Verma SB, Nayak S, Pasale RK, Kittner T, Wollina U. Late complication after tropic storm accident: subcutaneous and intracranial actinomycetoma. Int Wound J. 2008 Dec;5(5):655-9.
17. Vazquez Guillamet LJ, Malinis MF, Meyer JP. Emerging role of *Actinomyces meyeri* in brain abscesses: A case report and literature review. IDCases. 2017 Jul 20;10:26-29. doi: 10.1016/j.idcr.2017.07.007.
18. Pereira AJDSPR, Tavares AT, Prates M, Ribeiro N, Fonseca LF, Marques MDR, Proença F. Brain Abscess: A Rare Clinical Case with Oral Etiology. Case Rep Infect Dis. 2022 Jan 4;2022:5140259. doi: 10.1155/2022/5140259.
19. Funakoshi Y, Hatano T, Ando M, Chihara H, Takita W, Tokunaga K, Hashikawa T, Kamata T, Higashi E, Nagata I. Intracranial Subdural Abscess Caused by *Actinomyces meyeri* Related to Dental Treatment: A Case Report. NMC Case Rep J. 2020 Jun 24;7(3):135-139. doi: 10.2176/nmccrj.cr.2019-0246.
20. Narayanan NS, Sheriff F, Boyce JM. Teaching NeuroImages: CNS actinomycosis in an immunocompetent patient. Neurology. 2012 Sep 4;79(10):e85. doi: 10.1212/WNL.0b013e3182684623.
21. Chen KH, Lin CH. Brain abscess as an initial presentation in a patient of hereditary haemorrhagic telangiectasia caused by a novel ENG mutation. BMJ Case Rep. 2013 Feb 25;2013:bcr2013008802. doi: 10.1136/bcr-2013-008802.
22. Jain H, Singh G, Eranki A. *Actinomyces odontolyticus* causing meningitis and cervical abscess. Proc (Bayl Univ Med Cent). 2021 Apr 6;34(4):492-493. doi: 10.1080/08998280.2021.1907079.
23. Miller S, Walls T, Atkinson N, Zaleta S. A case of otitis media complicated by intracranial infection with *Actinomyces turicensis*. JMM Case Rep. 2014 Dec 1;1(4):e004408. doi: 10.1099/jmmcr.0.004408.
24. Hadgaonkar S, Rathi P, Vincent V, Shyam A, Sancheti P. Actinomycotic Infection of Spine - A Rare Disease with Diagnostic Challenge, an Update on Spinal Infection. J Orthop Case Rep. 2021;11(1):72-78. doi: 10.13107/jocr.2021.v11.i01.1970.
25. Stimac D, Jankovic D, Peric L, Anic K, Nimsky C. Intracerebral Abscess Caused by Actinomyces israelii. Cureus. 2020 Dec 13;12(12):e12058. doi: 10.7759/cureus.12058.
26. Yang WT, Grant M. Actinomyces neuii: a case report of a rare cause of acute infective endocarditis and literature review. BMC Infect Dis. 2019 Jun 10;19(1):511. doi: 10.1186/s12879-019-4149-2.
27. Clancy U, Ronayne A, Prentice MB, Jackson A. Actinomyces meyeri brain abscess following dental extraction. BMJ Case Rep. 2015 Apr 13;2015:bcr2014207548. doi: 10.1136/bcr-2014-207548.
28. Anderson IA, Jarral F, Sethi K, Chumas PD. Paediatric ventriculoperitoneal shunt infection caused by Actinomyces neuii. BMJ Case Rep. 2014 May 23;2014:bcr2014204576. doi: 10.1136/bcr-2014-204576.
29. Simpson AJ, Das SS, Mitchelmore IJ. Polymicrobial brain abscess involving Haemophilus paraphrophilus and Actinomyces odontolyticus. Postgrad Med J. 1996 May;72(847):297-8. doi: 10.1136/pgmj.72.847.297.
30. Roopesh Kumar VR, Madhugiri VS, Gundamaneni SK, Verma SK. Actinomycotic osteomyelitis of the cranial vault presenting with headache: an unusual presentation. BMJ Case Rep. 2014 Nov 24;2014:bcr2013202501. doi: 10.1136/bcr-2013-202501.
31. Perez A, Syngal G, Fathima S, Sandkovsky U. *Actinomyces*causing a brain abscess. Proc (Bayl Univ Med Cent). 2021 Jul 22;34(6):698-700. doi: 10.1080/08998280.2021.1945354.
32. la Cerda-Vargas MF, Rangel JAC, Mata EM, Ramírez-Cárdenas A, Sandoval-Bonilla BA. ^99m^Tc-UBI 29-41 bone SPECT/CT scan in craniofacial *Actinomyces israelii*: Misdiagnosis of cranial bone tumor - A case report. Surg Neurol Int. 2020 Dec 16;11:442. doi: 10.25259/SNI_684_2020.
33. Kapmaz M, Gülşen I, Kış N, Başaran S, Oksüz L, Gürler N. A Highly Rare Cause of Lumbar Spondylodiscitis with Epidural Abscess: Actinomyces israelii. Case Rep Infect Dis. 2014;2014:469075. doi: 10.1155/2014/469075. Epub 2014 Jun 12.
34. Cano EJ, Corsini Campioli C, Rodriguez AE, Enzler MJ. New-onset seizures and a tangled up Gram's stain: Actinomyces brain abscess. IDCases. 2020 Dec 11;23:e01024. doi: 10.1016/j.idcr.2020.e01024.
35. Park SH, Lee SW, Kang DH, Hwang JH, Sung JK, Hwang SK. The role of f-fluorodeoxyglucose positron emission tomography in the treatment of brain abscess. J Korean Neurosurg Soc. 2011 May;49(5):278-83. doi: 10.3340/jkns.2011.49.5.278. Epub 2011 May 31.
36. Bellingan GJ. Disseminated actinomycosis. BMJ. 1990 Dec 8;301(6764):1323-4. doi: 10.1136/bmj.301.6764.1323.
37. Na KY, Jang JH, Sung JY, Kim YW, Park YK. Actinomycotic brain abscess developed 10 years after head trauma. Korean J Pathol. 2013 Feb;47(1):82-5. doi: 10.4132/KoreanJPathol.2013.47.1.82. Epub 2013 Feb 25.
38. Park HJ, Park KH, Kim SH, Sung H, Choi SH, Kim YS, Woo JH, Lee SO. A Case of Disseminated Infection due to Actinomyces meyeri Involving Lung and Brain. Infect Chemother. 2014 Dec;46(4):269-73. doi: 10.3947/ic.2014.46.4.269. Epub 2014 Dec 29.
39. Funaki B, Rosenblum JD. MR of central nervous system actinomycosis. AJNR Am J Neuroradiol. 1995 May;16(5):1179-80. PMID: 7639151; PMCID: PMC8337809.
40. Honda H, Bankowski MJ, Kajioka EH, Chokrungvaranon N, Kim W, Gallacher ST. Thoracic vertebral actinomycosis: Actinomyces israelii and Fusobacterium nucleatum. J Clin Microbiol. 2008 Jun;46(6):2009-14. doi: 10.1128/JCM.01706-07. Epub 2008 Mar 12.
41. Kim DM, Kim SW. Destruction of the C2 Body due to Cervical Actinomycosis: Connection between Spinal Epidural Abscess and Retropharyngeal Abscess. Korean J Spine. 2017 Mar;14(1):20-22. doi: 10.14245/kjs.2017.14.1.20. Epub 2017 Mar 31
42. Fadda GL, Gisolo M, Crosetti E, Fulcheri A, Succo G. Intracranial complication of rhinosinusitis from actinomycosis of the paranasal sinuses: a rare case of abducens nerve palsy. Case Rep Otolaryngol. 2014;2014:601671. doi: 10.1155/2014/601671. Epub 2014 Aug 21.
43. Hagiya H, Otsuka F. Actinomyces meyeri meningitis: the need for anaerobic cerebrospinal fluid cultures. Intern Med. 2014;53(1):67-71. doi: 10.2169/internalmedicine.53.0403.
44. Patil VR, Joshi AR, Joshi SS, Patel D. Lumbosacral actinomycosis in an immunocompetent individual: An extremely rare case. J Craniovertebr Junction Spine. 2014 Oct;5(4):173-5. doi: 10.4103/0974-8237.147088.
45. Watkins RR, Anthony K, Schroder S, Hall GS. Ventriculoperitoneal shunt infection caused by Actinomyces neuii subsp. neuii. J Clin Microbiol. 2008 May;46(5):1888-9. doi: 10.1128/JCM.02141-07. Epub 2008 Mar 26.
46. Holland NR, Deibert E. CNS actinomycosis presenting with bilateral cavernous sinus syndrome. J Neurol Neurosurg Psychiatry. 1998 Jan;64(1):4. doi: 10.1136/jnnp.64.1.4.
47. Shen JY, Futran ND, Sardesai MG. Craniofacial *Actinomyces* osteomyelitis evolving from sinusitis. Radiol Case Rep. 2017 Dec 20;13(1):104-107. doi: 10.1016/j.radcr.2017.10.018.
48. Kobayashi T, Ford B, Fujita N, Appenheimer AB. Ocular Actinomycosis Mimicking Meningioma. Open Forum Infect Dis. 2020 May 19;7(6):ofaa170. doi: 10.1093/ofid/ofaa170.
49. Habib S, Siddiqui AH, Azam M, Siddiqui F, Chalhoub M. Actinomyces viscosus causing disseminated disease in a patient on methotrexate. Respir Med Case Rep. 2018 Aug 25;25:158-160. doi: 10.1016/j.rmcr.2018.08.009.
50. Grach SL, Tande AJ. A sticky situation: a case of *Actinomyces viscosus* vertebral osteomyelitis. J Bone Jt Infect. 2020 Sep 9;6(2):39-42. doi: 10.5194/jbji-6-39-2020.
51. Puzzilli F, Salvati M, Ruggeri A, Raco A, Bristot R, Bastianello S, Lunardi P. Intracranial actinomycosis in juvenile patients. Case report and review of the literature. Childs Nerv Syst. 1998 Sep;14(9):463-6.
52. King AD, Chan YL, Wong KS, Sung JJ, Fung K, Poon WS. Cranial actinomycosis. Singapore Med J. 1998 Oct;39(10):465-7. PMID: 9885710.
53. Ryu J, Lee KM. Intracranial Mycotic Aneurysm in a Patient with Abdominal Actinomycosis. World Neurosurg. 2021 Mar;147:161-163.
54. Gaïni S, Røge BT, Pedersen C, Pedersen SS, Brenøe AS. Severe Actinomyces israelii infection involving the entire spinal cord. Scand J Infect Dis. 2006;38(3):211-3
55. Olah E, Berger C, Boltshauser E, Nadal D. Cerebral actinomycosis before adolescence. Neuropediatrics. 2004 Aug;35(4):239-41.
56. Sharma BS, Banerjee AK, Sobti MK, Kak VK. Actinomycotic brain abscess. Clin Neurol Neurosurg 1990;92:373-6.
57. Ravindra N, Sadashiva N, Mahadevan A, Bhat DI, Saini J. Central Nervous System Actinomycosis-A Clinicoradiologic and Histopathologic Analysis. World Neurosurg. 2018 Aug;116:e362-e370.
58. Akhaddar A, Elouennass M, Baallal H, Boucetta M. Focal intracranial infections due to Actinomyces species in immunocompetent patients: diagnostic and therapeutic challenges. World Neurosurg. 2010 Aug-Sep;74(2-3):346-50.
59. Jamjoom AB, Jamjoom ZA, al-Hedaithy SS. Actinomycotic brain abscess successfully treated by burr hole aspiration and short course antimicrobial therapy. Br J Neurosurg. 1994;8(5):545-50.
60. Fabbri G, Guardigni V, Sarubbo S, Cultera R, Contini C (2014) Brain Abscess Sustained by *Actinomyces meyeri* in an Immunocompetent Patient. J Neurol Neurophysiol 5: 184.
61. Budenz CL, Tajudeen BA, Roehm PC. Actinomycosis of the temporal bone and brain: case report and review of the literature. Ann Otol Rhinol Laryngol. 2010 May;119(5):313-8.
62. Tsai MS, Tarn JJ, Liu KS, Chou YL, Shen CL. Multiple actinomyces brain abscesses: case report. J Clin Neurosci. 2001 Mar;8(2):183-6. doi: 10.1054/jocn.1999.0744. PMID: 11243774.
63. Lad SD, Chandy MJ. Cranio-facial actinomycosis. Br J Neurosurg. 1991;5(4):361-70. doi: 10.3109/02688699109002863. PMID: 1786131.
64. Navas E, Martínez-San Millán J, García-Villanueva M, de Blas A. Brain abscess with intracranial gas formation: case report. Clin Infect Dis. 1994 Jul;19(1):219-20.
65. Lagunes L, Len O, Sandiumenge A, Martínez-Saez E, Pumarola T, Bodro M, Macías A, Silva JT, Nuvials FX, Charco R, Moreso F, Pont T. Successful multiple organ donation after donor brain death due to Actinomyces israelii meningitis. Transpl Infect Dis. 2017 Aug;19(4).
66. Nugent NF, Murphy M, Kelly J. Scalp abscess--a cautionary tale. J Plast Reconstr Aesthet Surg. 2010 Aug;63(8):e619-21.
67. Mohindra S, Savardekar A, Rane S. Intracranial actinomycosis: varied clinical and radiologic presentations in two cases. Neurol India. 2012 May-Jun;60(3):325-7.
68. Mishra A, Prabhuraj AR, Bhat D, Nandeesh BN, Mhatre R. Intracranial Actinomycosis Manifesting as a Parenchymal Mass Lesion: A Case Report and Review of Literature. World Neurosurg. 2019 Feb;122:190-194
69. Ghobrial GM, Pisculli ML, Evans JJ, Bilyk JR, Farrell CJ. Odontogenic Sinusitis Resulting in Abscess Formation Within the Optic Chiasm and Tract: Case Report and Review. J Neuroophthalmol. 2016 Dec;36(4):393-398.
70. Opsomer D, Allaeys T, Alderweireldt AS, Baert E, Roche N. Intracranial complications of midline nasal dermoid cysts. Acta Chir Belg. 2019 Apr;119(2):125-128.
71. Soto-Hernández JL, Morales VA, Lara Giron JC, Balderrama Bañares J. Cranial epidural empyema with osteomyelitis caused by actinomyces, CT, and MRI appearance. Clin Imaging. 1999 Jul-Aug;23(4):209-14.
72. Haggerty CJ, Tender GC. Actinomycotic brain abscess and subdural empyema of odontogenic origin: case report and review of the literature. J Oral Maxillofac Surg. 2012 Mar;70(3):e210-3.
73. Koda Y, Seto Y, Takeichi S, Kimura H. Fatal subarachnoid hemorrhage complicating actinomycotic meningitis. Forensic Sci Int. 2003 Jul 8;134(2-3):169-71.
74. Rahiminejad M, Hasegawa H, Papadopoulos M, and MacKinnon A. Actinomycotic brain abscess. *BJR Case Rep* 2016; **2**: 20150370.
75. Imamura K, Kamitani H, Nakayasu H, Asai Y, Nakashima K. Purulent meningitis caused by Actinomyces successfully treated with rifampicin: a case report. Intern Med. 2011;50(10):1121-5.
76. Chotmongkol V, Panthavasit J, Chuesakoolvanich K. Actinomycotic meningitis: report of a case. J Med Assoc Thai. 2002 Jun;85(6):739-41.
77. Adeyemi OA, Gottardi-Littell N, Muro K, Kane K, Flaherty JP. Multiple brain abscesses due to Actinomyces species. Clin Neurol Neurosurg. 2008 Sep;110(8):847-9
78. Saleem MA, Ul Abideen Z, Kiani IS, Yousaf A, Rasheed A, Shabbir RW. Sporadic actinomycosis of the hip complicated by Central Nervous System infection. J Pak Med Assoc. 2017 Apr;67(4):637-640.
79. Ohta S, Nishizawa S, Namba H, Sugimura H. Bilateral cavernous sinus actinomycosis resulting in painful ophthalmoplegia. Case report. J Neurosurg. 2002 Mar;96(3):600-2.
80. Limaye HS, A Hinduja AR, Verma M, Oak PJ. A Case of CNS Actinomycosis: Rarer than Rare! Neurol India. 2021 Mar-Apr;69(2):475-477.
81. Ushikoshi S, Koyanagi I, Hida K, Iwasaki Y, Abe H. Spinal intrathecal actinomycosis: a case report. Surg Neurol. 1998 Sep;50(3):221-5.
82. Mehmet Sah İpek, Erdal Ozbek, Dilek Guldemir, Nezahat Akpolat. Neonatal Meningitis Caused by Actinomyces: A Case Report of the Most Probably New Strain. J Pediatr Infect Dis 2017; 12(02): 138-141
83. Khare M, Wade Z, Gupta H, Bhardwaj L, Kumawat N, Ola V. A case of brain abscess caused by actinomyces mimicking glioma: A rare presentation. Indian J Case Reports. 2019;5(3):215-216
84. Tom, Trisha S.M. PharmD^*†^; Villeneuve, David PharmD^*^; Terriff, Colleen M. PharmD, BCPS (AQ ID), AAHIVE^*†^ Disseminated Actinomycosis With Multifocal Abscesses, Infectious Diseases in Clinical Practice: May 2010 - Volume 18 - Issue 3 - p 204-207
85. Takahashi M, Nakanishi Y, Hamada Y, Hoshimoto Y, Aoki J, Karakida K. A Case of Brain Abscess Caused by Actinomyces Cardiffensis and Parvimonas Micra. Tokai J Exp Clin Med. 2020 Dec 20;45(4):189-194.
86. Lubomski M, Dalgliesh J, Lee K, Damodaran O, McKew G, Reddel S. *Actinomyces* cavernous sinus infection: a case and systematic literature review. Pract Neurol. 2018 Oct;18(5):373-377.
87. Dua RK, Bhat DI, Indira DB. Spinal actinomycosis: a rare disease. Neurol India. 2010 Mar-Apr;58(2):298-9.
88. Bouziri A, Khaldi A, Smaoui H, Menif K, Ben Jaballah N. Fatal subdural empyema caused by Streptococcus constellatus and Actinomyces viscosus in a child--case report. J Microbiol Immunol Infect. 2011 Oct;44(5):394-6.
89. Kumar M, Bajaj A, Tripathi M, Radotra BD, Tewari MK, Ahuja CK. Actinomyeces Cerebral Abscess Masquerading as Tuberculosis: Delayed Presentation following Head Trauma and Scalp Infection. Neurol India 2019;67:1123-6.
90. Douleh DG, Morone PJ, Johnson JE, Paueksakon P, Wellons JC III. Actinomycosis Mimicking Tolosa-Hunt Syndrome in a 6-Year-Old Boy: Case Report. Pediatr Neurosurg. 2016;51(4):214-7.
91. Hall WA. Hereditary hemorrhagic telangiectasia (Rendu-Osler-Weber disease) presenting with polymicrobial brain abscess. Case report. J Neurosurg. 1994 Aug;81(2):294-6.
92. Adler CH, Hurtig HI, Jacobson MP, Russin VL, Brooks ML. A woman with lung and brain masses. J Neuroimag 1992;2:97-101
93. T Wang, H Zhu, P Chen, et al. Intraspinal actinomycosis: a rare case report and literature review. Int J Clin Exp Med 2017;10(8):12698-12704.
94. K Harshith, K Nagarajan, A Sadayandi, Ra Debasis, et al. Transcalvarial and Transdural Involvement of Skull Actinomycosis with Recurrence. Indian J Neurosurg 2019;8:185–187
95. Vikas C. Spinal actinomycosis. J NTR Univ Health Sci 2014;3:122-4.

**Supplemental-5**

**Table 1: Other Microorganisms isolated in CNS Actinomycosis**

| **Microorganisms Number of Cases** |
| --- |
| *Fusobacterium* species 14  *Streptococcus intermedius* 6  *Streptococcus mitis* 4  *viridans group streptococci* (VGS) 4  *Staphylococcus epidermidis* 3  *Propionibacterium* 3  *Peptostreptococcus* 2  *S*t*aphylococcus haemolyticus*  2  *Coagulase-negative staphylococci (CoNS)*  2  *Escherichia coli* 2  *Aggregatibacter actinomycetemcomitans* 1  *Bacteroides species* 1  *Pseudomonas species* 1  *Proteus* 1 |

**Supplemental-6**

**Table 2:**

**The impact of polymicrobial infections on clinical outcome in CNS actinomycosis**

| **Variables** | **Polymicrobial infection** | **Monomicrobial infection** | **p-value** |
| --- | --- | --- | --- |
| Mortality | 3/35 (8.6%) | 10/83 (12%) | 0.42 |
| Neurological sequelae | 6/24 (25%) | 12/55 (21.8%) | 0.48 |
| Relapse | 1/25 (4%) | 2/46 (4.3%) | 0.71 |
| Mean duration of treatment (days) | 202±34 | 136±49 | 0.02 |
| Combination antibiotic use | 10/34 (29.4) | 16/70 (22.8%) | 0.51 |
